# Cannabinoid Receptor Type 2 Agonist JWH-133 Stimulates Antiviral Factors and Decreases Proviral, Inflammatory, and Neurotoxic Proteins in HIV-Infected Macrophage Secretome

**DOI:** 10.3390/ijms262110596

**Published:** 2025-10-30

**Authors:** Lester J. Rosario-Rodríguez, Yadira M. Cantres-Rosario, Ana E. Rodríguez De Jesús, Alana M. Mera-Pérez, Eduardo L. Tosado-Rodríguez, Abiel Roche Lima, Loyda M. Meléndez

**Affiliations:** 1 Department of Internal Medicine, University of Puerto Rico-Medical Sciences Campus, San Juan, PR 00936, USA; lester.rosario@upr.edu; 2 Translational Proteomics Center, Research Capacity Core, Center for Collaborative Research in Health Disparities (CCRHD), Academic Affairs Deanship, University of Puerto Rico-Medical Sciences Campus, San Juan, PR 00936, USA; yadira.cantres@upr.edu (Y.M.C.-R.); ana.rodriguez48@upr.edu (A.E.R.D.J.); 3 Department of Biology, University of Puerto Rico-Río Piedras Campus, San Juan, PR 00925, USA; alana.mera@upr.edu; 4 Integrated Informatics Services, Research Capacity Core, Center for Collaborative Research in Health Disparities (CCRHD), Academic Affairs Deanship, University of Puerto Rico-Medical Sciences Campus, San Juan, PR 00936, USA; tosadoe1@uagm.edu (E.L.T.-R.); abiel.roche@upr.edu (A.R.L.); 5 School of Dental Medicine, Universidad Ana G. Méndez, Gurabo, PR 00778, USA; 6 Department of Microbiology, University of Puerto Rico-Medical Sciences Campus, San Juan, PR 00936, USA

**Keywords:** HIV-associated neurocognitive disorders, CB2R, JWH-133, secretome, LC/MS/MS, TMT, proteomics

## Abstract

Although antiviral therapy has improved quality of life, around 50% of people with HIV (PWH) experience neurodegeneration and cognitive decline. This is prompted in part by the migration of HIV-infected monocyte-derived macrophages (MDMs) to the brain, leading to neuronal death. Previous studies in our lab have shown that HIV-infected MDMs secrete cathepsin B (CATB), which is a pro-inflammatory neurotoxic enzyme that is reduced by the addition of cannabinoid receptor-2 (CB2R) agonist JWH-133 to cell cultures. In this study, we aimed to identify the proteins secreted (secretome) by HIV-infected macrophages exposed to JWH-133 and quantify them using tandem mass tag (TMT) mass spectrometry. Frozen 13-day MDM supernatants from (1) an MDM negative control; (2) HIV+MDM, and (3) HIV+MDM-JWH-133 were compared in triplicate by mass spectrometry (LC/MS/MS) and analyzed for protein identification. Subsequently, the same samples were labeled by TMT labeling and quantified by LC/MS/MS. After a database search, 528 proteins were identified from all groups. Thereafter, proteins with more than three unique peptides and more than 10% coverage were selected for protein identification. Venn diagrams revealed one unique protein secreted by MDM-HIV, 10 unique proteins in HIV+MDM-JWH-133, and 15 common proteins in the three groups. CATB was unique to HIV+MDM. HIV+MDM exposed to JWH-133 showed proteins related to metabolism, cell organization, antiviral activity, and stress response. TMT analysis revealed 1454 proteins with abundance for statistical analysis based on FC ≥ |1.5| and *p*-value ≤ 0.05, of which Ruvb-like 1 and Hornerin decreased significantly with JWH-133 treatment. Both proteins stimulate HIV replication. In addition, HIV infection upregulated proteins associated with pathways of viral latency that were inhibited by JWH-133. In conclusion, JWH-133 treatment in HIV-infected macrophages leads to the secretion of antiviral host factors and decreases the secretion of proviral, inflammatory, and neurotoxic host factors.

## 1. Introduction

Approximately 40.8 million people were living with HIV worldwide in 2024 [1]. Even though new infections have decreased by 61% since the peak in 1996, comorbidities such as HIV-associated neurocognitive disorders (HAND) remain prevalent in approximately 50% of people with HIV (PWH) receiving combined antiretroviral therapy (cART) [1,2]. HIV-associated neurocognitive disorders (HAND) encompass a spectrum of cognitive dysfunction that ranges from asymptomatic neurocognitive impairment (ANI) to mild neurocognitive disorder (MND) and HIV-associated dementia (HAD). These neuropathological manifestations impact episodic and working memory, attention/concentration, executive functioning, language processing, and visuospatial/perceptual–motor skills. As the severity progresses, deficits extend to impairments in activities of daily living. Behavioral changes, confusion, depression, anxiety, and movement disorders are some of the symptoms of HAND [3,4]. HAND are characterized by neurodegeneration that is triggered by early HIV infection of the brain and chronic inflammation due to continuous low-level viral replication.

The HIV-1 replication cycle in immune cells is classically divided into two distinct phases: the early (pre-integration) and late (post-integration) stages. The early phase involves molecular events initiating with virion attachment to host cell surface receptors, primarily CD4 and co-receptors such as CCR5 or CXCR4, followed by membrane fusion and viral entry. Subsequent steps include the reverse transcription of the single-stranded viral RNA genome into double-stranded DNA via the viral reverse transcriptase, disassembly of the viral capsid (uncoating), translocation of the pre-integration complex into the nucleus, and integration of the proviral DNA into the host chromatin, mediated by the viral integrase enzyme.

The late phase consists of proviral transcription and culminates in the production of infectious progeny virions. This includes the transcription of integrated proviral DNA by host RNA polymerase II, nuclear export of spliced and unspliced viral transcripts, and translation of these RNAs into structural (Gag, Gag-Pol), envelope (Env), and regulatory/accessory proteins. The Gag and Gag-Pol polyproteins, along with Env glycoproteins, are trafficked to the plasma membrane, where virion assembly occurs. Genomic RNA is selectively packaged into assembling particles via Gag-mediated encapsulation. Env incorporation and membrane curvature lead to the budding of immature virions, which subsequently undergo protease-mediated maturation to yield infectious HIV-1 particles [5]. However, HIV-1 replication in macrophage reservoirs is mostly initiated and sustained by R5-tropic viral strains, as X4-tropic strains promote the depletion of macrophages [6]. HIV-infected myeloid cells and T cells enter the CNS during HIV infection and become a source of viral replication in the brain [7,8,9,10]. These cells, including resident HIV-infected microglia, can secrete viral proteins or cellular toxins that can affect the neurons, causing toxicity. One of the secreted proteins by macrophages and microglia is cathepsin B (CATB), a lysosomal enzyme that can induce neurotoxicity and inflammation [11,12,13,14,15,16,17,18,19].

Our group has previously reported increased secretion and neurotoxicity by HIV-infected macrophages in culture, which can both be reduced by exposure to cannabinoid receptor type 2 (CB2R) agonist JWH-133 [15,16]. In addition, we demonstrated that CB2R activation decreases HIV-1 replication, supporting several studies [16,20,21,22,23]. At the intracellular level, HIV-infected macrophages treated with JWH-133 downregulated the expression of CATB and proteins associated with NF-*κ*B signaling, the Nrf2-mediated oxidative stress response, and lysosomal exocytosis [15]. Cannabinoid receptor-2 has shown therapeutic potential as a target against HAND and other CNS disorders, such as Alzheimer’s disease (AD), amyotrophic lateral sclerosis (ALS), Parkinson’s disease (PD), multiple sclerosis (MS), Huntington’s disease (HD), neuropathic pain, and migraine [24]. In this study, we hypothesized that the secretome of JWH-133-exposed HIV-infected macrophages contained protective factors against infection, inflammation, and neurotoxicity. We first identified the proteins present in the secretome from monocyte-derived macrophages (MDMs) infected with HIV-1 and exposed to the CB2R agonist compared to controls by LC MS/MS. Thereafter, we applied TMT quantitative proteomics and found that CB2R activation by JWH-133 reduced the secretion of inflammatory proteins including CATB and proteins related to HIV replication, while it stimulated the secretion of proteins involved in cell metabolism, which are beneficial for cell protection in HIV-MDM. Therefore, CB2R agonists could benefit the surrounding cells and tissues, making them potential supplements to antiretroviral therapy.

## 2. Results

### 2.1. HIV Stimulates the Secretion of CATB by HIV-MDM, While JWH-133 Stimulates the Secretion of Proteins Related to Metabolism, Cell Organization, and Antiviral and Stress Responses

**Figure 1:**
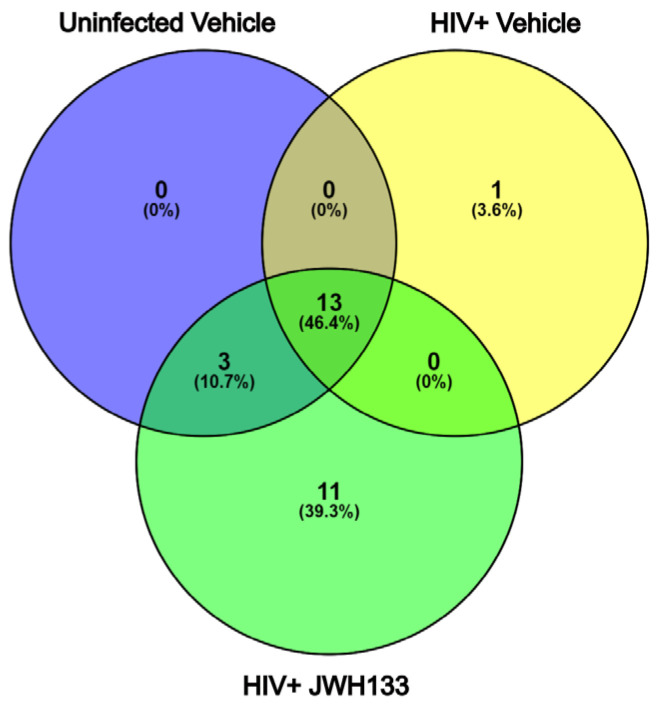
Venn diagram of protein identification from HIV-infected MDM secretome exposed to JWH-133. From 528 total proteins identified in the three groups in triplicate, those with more than 3 unique peptides and more than 10% sequence coverage were selected for protein identification. Only proteins found common to each triplicate group were selected for comparison between the groups. Of these, one unique protein was found in HIV-infected MDMs, while 11 unique proteins were found in HIV-infected MDMs exposed to the CB2R agonist JWH-133. Thirteen (13) proteins were found common to the three groups.

**Figure 2:**
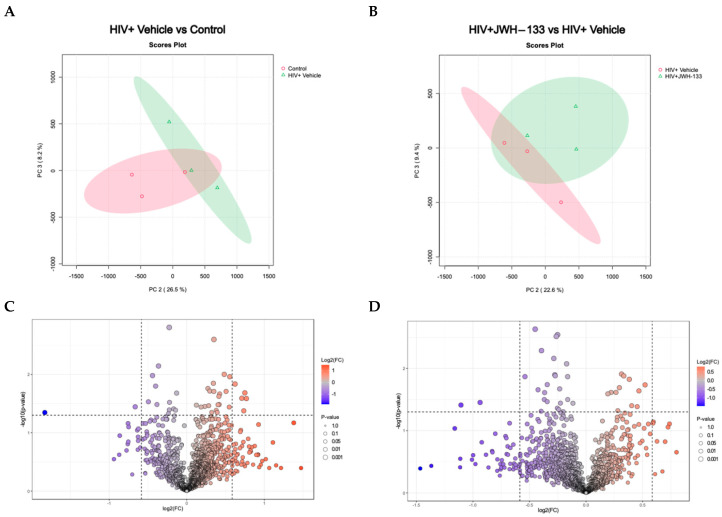
Differentially expressed proteins in HIV-MDM samples exposed to JWH-133 vs. controls. A 2D principal component analysis (PCA) comparing HIV+ vehicle vs. control samples (uninfected vehicle), using the protein concentration as the evaluating variable (**A**). The PCA comparing HIV+JWH-133 vs. HIV+ vehicle samples, using the protein concentration as the evaluating variable. The ellipses indicate that all samples within are related with 95% confidence. PERMANOVA *p*-value (based on 999 permutations) = 0.2. (**B**). Volcano plot for HIV+ vehicle vs. control comparison depicting differentially abundant proteins with a fold change ≥ |1.5| and *p*-value ≤ 0.05. (**C**) Volcano plot for HIV+JWH-133 vs. HIV+ vehicle comparison depicting differentially abundant proteins with a fold change ≥ |1.5| and *p*-value ≤ 0.05 (**D**).

In our previous studies, we have demonstrated that macrophages with increased CATB secretion after HIV infection exert CATB-mediated neurotoxicity [3,4,5], while macrophages with decreased CATB secretion after HIV infection are unable to promote neurotoxicity [12,16]. Subsequently, we demonstrated that JWH-133 treatment decreased CATB levels in HIV-infected MDMs [16]. In a representative number of HIV-infected MDM cells (*n* = 3) with increased CATB secretion and treated with JWH-133, we showed that this CB2R agonist caused the downregulation of intracellular pathways involving oxidative stress, lysosomal exocytosis, and NF-κB signaling [15]. In this study, we further analyzed the secretome of representative HIV-infected MDMs exposed to JWH-133 compared to controls in triplicate and identified 528 total proteins in the three groups. Those with more than three unique peptides and more than 10% sequence coverage were included for protein identification (Supplementary Tables S1–S3). Only proteins found common to each triplicate group were selected for comparison between the groups (Supplementary Figures S1–S3). Of these, only one unique protein was found in HIV-infected MDMs (Figure 1). This was CATB, which has been consistently demonstrated to be secreted in our previous studies and to induce neurotoxicity [11,12,14,15,16,17,18,19] (Table 1; Supplementary Tables S2, S4 and S5). Eleven (11) unique proteins were found in HIV-infected MDMs exposed to the CB2R agonist JWH-133 (Figure 1). Most of these proteins identified were beneficial for cell protection during glycolysis (PGK1, GAPDH), lipid production (PGD), cytoskeletal rearrangement (MSN, ACTN1, ACTR3), protection from oxidative stress (HSPA8), and neuronal regeneration (CTSS) (Table 2; Supplementary Tables S3–S5). Thirteen (13) proteins were found common to the three groups (Figure 2; Supplementary Tables S3–S5). To determine if these or additional proteins were differentially expressed between the groups, we proceeded with tandem mass tag (TMT) quantitative proteomics.

### 2.2. HIV Infection and JWH−133 Treatment in HIV-Infected MDMs Lead to a Unique Profile of Differentially Expressed Proteins in Supernatants

To identify differentially expressed proteins secreted by MDMs exposed to CB2R, we applied TMT quantitative proteomics. A total of 1527 proteins were processed before the statistical analysis. Of these, we analyzed 1454 proteins with abundance values available for bioinformatics analyses. The statistical analysis performed was a one-factor analysis between experimental samples and controls. Statistical analysis revealed differentially abundant proteins between groups based on FC ≥ |1.5| and *p*-value ≤ 0.05. We used normalized abundances [25] derived from Proteome Discoverer (Thermo Fisher, Waltham, MA, USA). No data imputation was performed with MetaboAnalyst, as the input data had no missing values. No data transformation, normalization, or scaling was performed in MetaboAnalyst, as the input data were previously scaled in Proteome Discoverer (Supplementary Table S6).

Principal component analysis (PCA) is a crucial asset in proteomics analyses, serving as an invaluable tool for streamlining complex datasets through dimensionality reduction and pattern recognition. In proteomics investigations, where the data intricacy and volume are notably high, the significance of PCA lies in its capacity to distinguish pertinent information and reveal latent trends. This methodology becomes indispensable in extracting meaningful insights from the extensive data generated by mass spectrometry and other advanced techniques. By condensing the dataset into a concise array of orthogonal components, PCA facilitates the visual representation of protein relationships and assists in pinpointing pivotal features influencing biological variations. This analytical approach proves instrumental in navigating the intricacies of proteomic landscapes, offering a clearer perspective and contributing to a greater understanding of underlying biological mechanisms. The PCA analysis of MDM-HIV-vehicle vs. MDM controls revealed similarities within replicates of the same group and differences between the two groups (Figure 2A). Likewise, similarities within the replicates and differences between the two groups of MDM-HIV-vehicle and HIV-JWH-133 were observed (Figure 2B).

Volcano plots were prepared with the data that met the significance criteria of fold change ≥ |1.5| and *p*-value ≤ 0.05 to visualize the number of differentially expressed proteins between MDM-HIV-vehicle and MDM controls (Figure 2C) and between MDM-HIV vehicle and HIV-JWH-133 (Figure 2D). Nine (*n* = 9) differentially abundant proteins were found between all groups (Table 3). In the HIV+ vehicle vs. control group, five proteins were more abundant, while two were less abundant. In the supernatants of HIV-infected MDMs exposed to JWH-133, only two proteins were differentially less abundant.

An analysis of the differentially abundant proteins in HIV+MDM vs. MDM control supernatants revealed that the five more abundant proteins in HIV+MDM were associated with inflammatory processes (Q96S97), glutamine–glutamate circulation, the synthesis of proteins and cell growth (Q96QD8), antigen processing and presentation (Q6P179), sugar metabolism (Q961J6), and calcium-dependent vesicle fusion (O75923). Meanwhile, the two differentially less abundant proteins in HIV+MDM were associated with the Src signaling pathway, B cell and macrophage adhesion, cytoskeletal organization (O75563), and the dynein transport machinery for microtubule motility and cellular trafficking (Q5VUJ9) (Table 4).

Two differentially expressed proteins secreted by HIV-infected macrophages exposed to JHW-133 were less abundant compared to unexposed HIV+MDM. These included two important proteins for antimicrobial activity that were both downregulated. The first, RVBL1, is a component of several important host protein complexes involved in DNA repair and gene regulation. RVBL1/2 is indispensable for the pro-inflammatory response and the antimicrobial activity of macrophages. The other protein, Hornerin (HRNR), an S100 protein, is involved in calcium binding and pathological cell functions (Table 5).

### 2.3. Proteomics Pathway Analysis of Differentially Expressed Proteins Reveals That HIV Infection Upregulated the Secretion of Inflammatory and Neurotoxic Pathways (e.g., NF-κB, MHC Class I Processing), Whereas JWH-133 Treatment Downregulated These Responses

**Figure 3:**
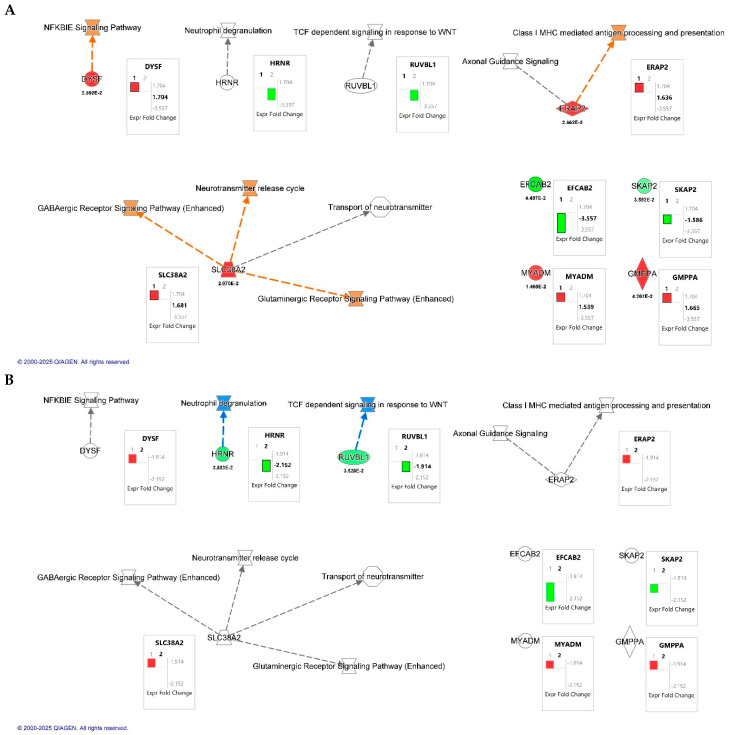
Canonical pathway overlay analysis of differentially expressed proteins identified by quantitative proteomics in HIV-infected macrophages. Canonical pathway maps were generated using Ingenuity Pathway Analysis (IPA; QIAGEN Inc., Redwood City, CA, USA). (**A**) Comparison of HIV+MDM-vehicle vs. control. (**B**) Comparison of HIV+MDM- JWH-133 vs. HIV+MDM-vehicle macrophages. Networks highlight molecular interactions and biological pathways affected by HIV infection and CB2R agonist treatment, including NF-κB inhibitor epsilon (NFKBIE) signaling, GABAergic and glutamatergic receptor signaling, class I MHC-mediated antigen processing and presentation, and the neurotransmitter release cycle. Box plots next to each node display the log_2_ fold change (FC) values for individual proteins, with *p*-values shown below the symbols for significant results. The black bars labeled “1” or “2” within the fold change boxes denote the dataset used for the overlay, corresponding to Dataset 1 (HIV+MDM-vehicle vs. control) and Dataset 2 (HIV+MDM JWH-133 vs. HIV+MDM-vehicle), respectively. Orange and red nodes indicate upregulated proteins or pathways, while green and blue nodes represent downregulation relative to the comparison group.

Canonical pathway overlay using Ingenuity Pathway Analysis (IPA; QIAGEN Inc., Redwood City, CA, USA) revealed key signaling pathways modulated by HIV infection and CB2R activation in macrophages (Figure 3). A total of seven dysregulated proteins were included in the analysis. In the HIV+MDM vehicle vs. control comparison (Figure 3A), upregulated pathways involved NF-κB inhibitor epsilon (IκBε), a key inhibitor of NF-κB pathway signaling, GABAergic and glutaminergic receptor signaling, and class I MHC-mediated antigen processing and presentation. Conversely, treatment with JWH-133 (Figure 3B) attenuated these same pathways. IPA functional annotation further categorized the seven dysregulated proteins according to their cellular localization and molecular function (Table 6). In HIV+MDM vehicle vs. control, most proteins were localized to the cytoplasm or plasma membrane, participating in membrane dynamics, protein processing, and immune signaling, including DYSF, SLC38A2, and SKAP2. Cytoplasmic enzymes such as ERAP2 and GMPPA were linked to antigen processing and glycoprotein biosynthesis, while MYADM (nuclear) was associated with myeloid differentiation. In contrast, the HIV+MDM-JWH-133 vs. HIV+MDM vehicle comparison identified only two significant abundant proteins: HRNR (cytoplasmic; stress response/structural) and RUVBL1 (nuclear; transcriptional regulator), suggesting a transition toward transcriptional regulation and structural stabilization following CB2R activation. Collectively, these findings indicate that HIV infection primarily affects cytoplasmic and membrane-associated inflammatory and metabolic pathways, whereas JWH-133 treatment modulates nuclear and regulatory proteins, contributing to cellular protection and homeostatic balance.

## 3. Discussion

HIV-associated neurocognitive disorders remain prevalent despite many years of combined antiretroviral therapy [3,4]. Combined approaches are needed to decrease the neuroinflammation and neurotoxicity induced by HIV-infected cells. CB2Rs are present in brain microglia and other tissues, mediating the immunosuppressive and anti-inflammatory effects of endocannabinoids [24]. CB2R agonists could play an important role in ameliorating HAND and other neuroinflammatory and neurodegenerative diseases, including AD, Parkinson’s, MS, ALS, and Huntington’s [24]. Unlike CB1R, targeting CB2R offers therapeutic benefits without psychotropic effects, making it an attractive target in conditions requiring long-term treatment. The literature consisting of in vivo pathology studies of white matter from HIV patients with HIVE has revealed the increased expression of CB2R in microglia, astrocytes, and perivascular macrophages [40]. CB2R upregulation was reported in the cortical tissue of SIV-infected rhesus macaques, perivascular monocytes/macrophages, and microglia [41]. In the cited study, the pattern of CB1R expression was not modified in the brains of infected animals compared with control animals. CB2R expression was induced in activated microglial cells, associated with deposits of β-amyloid peptide in Alzheimer’s disease [42]. These results suggest that the endocannabinoid system may participate in the development of HIV-induced encephalitis.

Our studies have been mainly focused on in vitro human HIV-infected macrophage cell cultures [15,16]. Using this model, we have demonstrated that the CB2R agonist JWH-133 shows promising results by decreasing intracellular HIV-1 replication, inflammation, oxidative stress, and CATB expression and its secretion [16]. Furthermore, we have demonstrated that this CB2R agonist decreased HIV-induced neurotoxicity and CATB neurotoxic potential from infected MDM supernatants [16]. In searching for the intracellular mechanisms affected by CB2R activation with JWH-133 on HIV-MDM, cell lysates were analyzed using quantitative proteomics. We found that JWH-133 agonist treatment downregulated several proteins associated with NF-*κ*B signaling, the Nrf2-mediated oxidative stress response, and lysosomal exocytosis in HIV-MDM, warranting in vivo studies to test its potential against HAND [15].

In this study, we hypothesized that the proteome of HIV-infected macrophage supernatants (secretome) exposed to JWH-133 would contain protective factors against HIV-1 infection, inflammation, and neurotoxicity. We began with protein identification (ID) as a screening test to determine the number and types of proteins that could be identified in the supernatants of each group—HIV-infected MDM controls (vehicle-treated) or HIV-infected MDMs treated with JWH-133—according to the parameters established (three or more peptides, more than 10% sequence coverage). Once we had identified common and unique proteins in each group, we sought to quantify the differential expression of proteins between the experimental and control groups. This was achieved with quantitative proteomics and TMT labeling. TMT labeling proteomics led us to determine the differential expression of proteins present in all groups compared using the established statistical criteria in terms of fold changes and *p*-values. Each technique (protein ID vs. TMT) provided a different type of analysis. The first one provided protein IDs, as well as common and unique proteins in each group, visualizing them with Venn diagrams, while TMT provided protein quantitation of differentially expressed proteins between experimental and control samples. Each method provided a different window for comparisons. If we wish to determine whether both methods identified similar proteins, we could examine the 15 common proteins identified by protein ID (including albumin and IgG, which were not considered in the analyses). Of these, eight (8) were also present in the TMT experiments; however, these did not meet the significance criteria for differentially expressed proteins and were not included in the final analyses (Supplementary Table S7). TMT allowed us to determine the most significant differentially expressed proteins. We first identified unique and common proteins present in the secretome from MDMs infected with HIV-1 and exposed to the CB2R agonist compared to controls by LC MS/MS. Among the unique proteins secreted by HIV-MDM, we only detected immunoglobulin and CATB, which has been demonstrated in previous studies using ELISA [11,12,14,15,16,17,18,19]. Immunoglobulin is not secreted by macrophages, but its presence may indicate remaining serum immunoglobulin attached to the cells by Fc receptors by nonspecific binding. Eleven (11) unique proteins were found in HIV-infected MDMs exposed to the CB2R agonist JWH-133. Most of these secreted proteins identified were beneficial for cell protection during glycolysis (PGK1, GAPDH), lipid production (PGD), cytoskeletal rearrangement (MSN, ACTN1, ACTR3), protection from oxidative stress (HSPA8), and neuronal regeneration (CTSS). These results support our hypothesis that the CB2R agonist promotes the secretion of beneficial proteins to the surrounding cells, affecting oxidative stress pathways, as previously reported by our group [15]. Fifteen (15) proteins were identified as common to the three groups, and most of these proteins were related to cell organization and biogenesis. Different proteins were detected in HIV-MDM treated with JWH-133. Alpha-1-antitrypsin (SERPINA1) decreases HIV-1 replication in PBMCs and monocytes by reducing NF-κB activation [43,44]. SERPINA1 binds to gp41, blocks HIV-1 entry into CD4+ T cells and inhibits its replication by suppressing NF-κB activation through the alteration of IκBα ubiquitination [44,45,46]. The analysis of a clinical case indicates that individuals with pre-existing SERPINA1 deficiency experience faster progression of HIV/AIDS, implying that SERPINA1 may function as an endogenous suppressor of the disease [47,48]. PKG1 binds to HIV-1 Tat-Specific Factor 1, an essential host factor involved in the export of HIV RNAs from the nucleus to the cytoplasm, possibly contributing to the antiviral effects exerted by JWH-133 [49]. Hsc70 (HSP8) regulates HIV-mediated cell fusion and multinucleated giant cell formation in monocytes [50]. It also protects cardiomyocytes from oxidative stress [51]. Similarly, 6-phosphogluconate dehydrogenase (PGD) is part of the oxidative pentose phosphate pathway and is transcriptionally activated by Nrf2, leading to the production of NADPH and ROS clearance [52]. Soluble factors such as galectin-3 (IGALS3) released by activated HIV-infected T cells promote the downregulation of CD4 expression and HIV replication in macrophages through the modulation of protein kinase C and NF-kB signaling [53]. Therefore, the released galectin-3 from HIV-infected MDMs may contribute to the control of HIV infection and replication. Cathepsin S is an endosomal protease that participates in HIV-1 gp120 antigen processing [54]. Cathepsin S (CTSS) is required for major histocompatibility complex class II (MHC-II) maturation, contributes to extracellular matrix (ECM) remodeling, and modulates inflammatory responses [55]. Therefore, cathepsin S could be another factor contributing to HIV-1 infection control and the resolution of inflammation. Extracellular GAPDH interacts with cell adhesion molecule L1 and promotes neurite outgrowth [56]; therefore, it could aid in neuronal repair after HIV-induced damage. Cytoskeletal proteins were also identified. Arp3 (ACTR3) is necessary for HIV-1 env-mediated cell–cell fusion, virus–cell fusion, and HIV-1 infection in U87 cells expressing CD4 and CCR5 [57]. Moesin functions as a negative regulator of R5-tropic HIV-1 infection after the membrane fusion step [58], but, in T cells, HIV-1 infection increases its RNA expression [59]. These results imply that CB2R activation in HIV-infected MDMs could modulate viral cell-to-cell transmission.

To determine if these proteins were differentially expressed between the groups, we performed additional experiments with TMT quantitative proteomics. Analysis of the differentially abundant proteins in HIV+MDM vs. MDM control supernatants using TMT revealed that the five more abundant proteins secreted in HIV+MDM were associated with inflammatory processes, the transport of two proteins across membranes, antigen processing and presentation, phosphate and mannose metabolism, and calcium-dependent vesicle fusion. These are cellular functions that the viruses use for their replication. Meanwhile, the two less abundant proteins in HIV+MDM were associated with the Src signaling pathway, B cell and macrophage adhesion, cytoskeletal organization, the dynein transport machinery for microtubule motility, and cellular trafficking, which reduce MDM immune defenses.

Two differentially expressed proteins secreted by HIV-infected macrophages exposed to JHW-133 were less abundant compared to HIV+MDM. These included proteins important in antimicrobial activity, which were both downregulated. The first is RVBL1, a component of several important host protein complexes involved in DNA repair and gene regulation that is indispensable for pro-inflammatory responses and the antimicrobial activity of macrophages. In PWH, it has been reported that RVBL2 levels positively correlate with the HIV-1 viral load and disease progression status. These findings reveal a mechanism by which HIV-1 regulates RVBL2 protein expression [37]. While HIV-1 can hijack exosomal machinery for the trafficking of viral RNA and proteins, and HIV-infected cells can secrete exosomes containing viral components such as the HIV-Env protein, the specific role of RVBL1 or its secretion in exosomes has not been reported [38]. The other less abundant protein in supernatants from HIV-MDM exposed to JWH-133, Hornerin, is an S100 family protein involved in normal and pathological functions including inflammatory and immune responses, calcium homeostasis, cell differentiation, and death. Inhibitors of this protein are being considered as therapeutics for diabetes, heart disease, cancer, and neurological diseases. Therefore, the downregulation of these two proteins by CB2R results in decreased inflammation and could be beneficial for surrounding cells and tissues [39]. It has been reported in HIV studies that the cellular biotinylated Hornerin protein can be incorporated into HIV-1 Gag virus-like particles [39]. However, in searching the literature, we did not find any previous study that identified Hornerin as secreted by macrophages. In our previous study [15], we found that the intracellular expression of RuvB-like 1 (RVBL1) and Hornerin (HRNR) was increased by HIV and decreased by JWH-133 treatment. Supporting these previous findings, we observed that RVBL1 and HRNR secretion was reduced by JWH-133 treatment in HIV-infected MDM supernatants in the current study. Therefore, RVBL1 and HRNR represent potential targets against HIV-1 infection and inflammation. Future studies focused on manipulating the expression and/or secretion of these proteins to evaluate their impacts on HIV-1 replication and inflammation from macrophages are warranted.

To further understand the role of JWH-133 in the modulation of the differentially expressed proteins identified by TMT labeling proteomics, Ingenuity Pathway Analysis (IPA) was conducted (Figure 3). The results revealed that HIV infection upregulated proteins that are associated with HIV-infected MDMs transitioning to a latent reservoir (e.g., NF-κB inhibitor epsilon (IκBε), MHC class I processing and antigen presentation, GABAergic and glutaminergic receptor signaling, and the neurotransmitter release cycle, whereas JWH-133 treatment inhibited these pathways. As reviewed by Khan and collaborators [60], IκBε is abundantly expressed in cells comprising the latent HIV reservoir, where it retains the activating NF-κB subunits c-Rel and p65 in the cytoplasm. This cytoplasmic sequestration blocks their translocation to the nucleus and thereby inhibits the activation of the HIV long terminal repeat (LTR). Furthermore, its inhibition leads to HIV-1 activation in latently infected cells. Therefore, targeting IκBε has been proposed as an alternative strategy for HIV latency reversal. Therefore, our results suggest that increased dysferlin release by HIV-infected MDMs at day 13 pi could aid in the control of NF-κB activation through IκBε signaling to establish latent HIV-1 reservoirs by an unknown mechanism. This hypothesis is supported by studies that have demonstrated that HIV-infected MDMs enter a state of quiescence and latency after day 12 pi by reducing NF-κB p65, RNA polymerase II, and p-TEFb recruitment to the HIV-1 promoter [61]. The role of dysferlin in IκBε signaling and HIV latency should be explored in future studies. PWH under cART show elevated plasma levels of glutamate and its derivative, GABA. Moreover, monocytes express the GABA receptor, and its activation induces increased ROS production [62]. SLC38A2 (also known as SNAT2) is a glutamine transporter that, when overexpressed, induces resistance to oxidative stress in triple-negative breast cancer cell lines [63]. Beyond fatty acids and glucose as energy sources, latent HIV reservoirs depend on glutamine, glutamate, and alpha-ketoglutarate (α-KG) as key metabolic substrates [64]. Therefore, our findings suggest that, by day 13 pi, HIV-infected MDMs use glutamate/glutamine metabolism and activate GABAergic and glutaminergic receptors to establish a latent reservoir, whereas treatment with JWH-133 from day one post-infection prevented this metabolic shift by maintaining control of HIV-1 replication. This is also supported by the presence of proteins that participate in glycolysis in JWH-133 HIV-infected MDMs (Table 2). On the other hand, the high expression of SLC38A2 may lead to the increased release of the neurotransmitter glutamate, leading to neuronal excitotoxicity. In rhesus macaques, SIV infection leads to the upregulation of *SLC38A2* and *SLC7A11*—associated with excitotoxicity—in the basal ganglia, and the chronic administration of cannabinoids (THC) decreases their expression. Therefore, our results suggest that CB2R activation could modulate glutamine/glutamate-mediated excitotoxicity in HIV. HIV infection upregulated the expression of ERAP2, which is involved in MHC I class antigen presentation and CD8+ T cell activation. Studies have demonstrated that ERAP2 has antiviral activity in HIV-infected MDMs, reducing viral replication by activating MDMs, leading to the release of IL-1-beta, TNF-alpha, IL-6, and IL-8, as well as inflammasome activation and increased phagocytosis [65]. Furthermore, HIV infection downregulated the release of SKAP2 and DRC8, which can mediate HIV-1 latency reactivation and HIV-1 motility, respectively [34,35] (Table 4). Therefore, our findings support the hypothesis that HIV-infected MDMs transition to a latent phase in which the control of HIV-1 transcription is necessary to maintain the reservoir. Therefore, studies assessing the roles of dysferlin, SLC38A2, SKAP2, and ERAP2 in HIV-induced latency in MDMs are warranted.

Apart from the predicted mechanisms that were upregulated by HIV and inhibited by JWH-133 treatment, CB2R activation by JWH-133 reduced the expression of HRNR and RUVBL1, which could lead to the suppression of neutrophil degranulation and TCF-dependent signaling in response to WNT, respectively (Figure 3B). Chronic HIV infection could lead to neutrophil dysfunction, augmenting inflammation and promoting tissue damage [66]. Therefore, JWH-133 could prevent inflammatory responses by neutrophils by decreasing the availability of HRNR. Increased WNT signaling in macrophages reduces HIV-1 replication, possibly to establish HIV-1 latency [67]. Although HIV infection did not increase RUVBL1 and TCF-dependent signaling in response to WNT signaling to a significant level, our results suggest that JWH-133 treatment diminishes the availability of RUVBL1, reducing the potential for HIV-infected macrophages to activate WNT signaling and form latent reservoirs. Future studies assessing the effect of CB2R activation in HIV-1 latency are needed.

Recently, a group of investigators has reported pilot studies in humans using cannabinoids against HIV-induced replication and inflammation [68]. However, these have been performed with ∆9-tetrahydrocannabinol (THC) and cannabidiol (CBD), which are partial agonists of CB2R. These investigators reported non-significant changes in HIV RNA replication and inflammatory markers. Despite these findings, they concluded that oral cannabinoids showed promise in alleviating immune activation and decreasing T cell exhaustion and immune senescence. Approximately 5–10 selective CB2R agonists have been approved for human clinical trials. Although none of them have been approved for human use, lenabasum and olorinab have shown promise against inflammatory diseases, with minor to moderate adverse effects, such as nausea, headache, nasopharyngitis, dizziness, dry mouth, and upper respiratory tract infections, among others [24]. Clinical trials using CB2R agonists are imperative to determine their efficacy against HAND.

This study has some limitations. We only used female donors, because previous studies in our laboratory have shown that females secrete higher levels of cathepsin B than men [16], and higher levels of cathepsin B in HIV-infected MDM supernatants correlate with increased neurotoxicity [12]. Sex differences in CB2R expression and agonism have been reported in mice and rats, respectively [69,70,71,72]. In the human ageing heart, the expression of CB2R is higher in men than women [73]. Therefore, future studies evaluating sex differences in CB2R agonism against HIV replication and inflammation are warranted. Moreover, we evaluated the effects of a single synthetic agonist (JWH-133); therefore, future studies evaluating other CB2R agonists are needed. In addition, this work was limited to the use of an R5-tropic strain (HIV-1ADA). However, CCR5 is the principal co-receptor for the HIV infection of immune cells, since viral R5 strains are mostly responsible for the initial infection, whereas CXCR4-tropic strains only occasionally cause the initial infection [74,75]. Moreover, HIV-1ADA CCR5-tropic has been used in all our previous studies evaluating cathepsin B secretion and neurotoxicity. Highly cytopathic CXCR4-tropic virus strains were not considered for these studies because the HIV-1 infection of MDMs with CXCR4-tropic virus induces productive HIV-1 replication in MDMs until day 7 pi, and then it decreases drastically and becomes undetectable at day 10 pi due to viral-induced cell death [6]. Therefore, a highly cytopathic CXCR4-tropic virus will not allow us to maintain sufficient viable macrophages in culture until the timepoint at which HIV-infected macrophages begin to induce cathepsin B-induced neuronal death (after day 12pi), as demonstrated by a previous study in our laboratory [11]. Lastly, we acknowledge the omission of testing uninfected cells exposed to JWH-133 in this study as a limitation that will need to be addressed in future studies. The testing of JWH-133 in uninfected cells is necessary to rule out unwanted side effects and assess the different toxicities and tolerances of uninfected versus infected cells. However, we have previously tested the cell viability of uninfected cells treated with JWH-133 at different concentrations, with no significant effects for any of the concentrations tested by day 13 pi, including the concentration of 0.5 μM used for the current study [16].

## 4. Materials and Methods

### 4.1. Macrophage Isolation, HIV-1ADA Infection, Treatments with JWH-133 Agonist, and Collection of Serum-Free Supernatants

**Figure 4:**
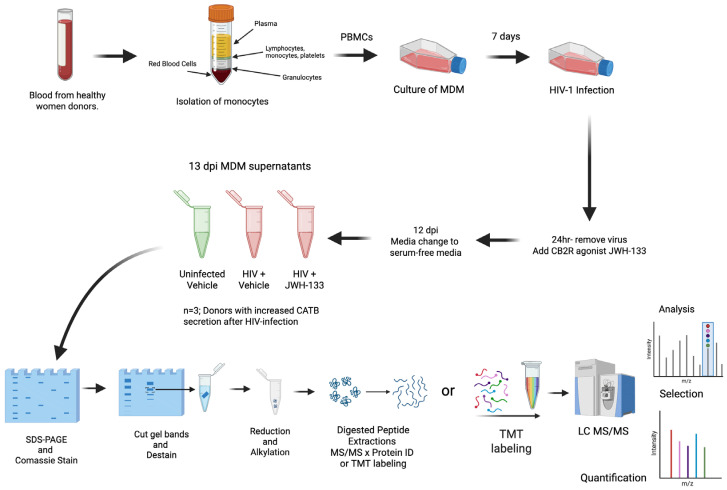
Diagram of experimental design focusing on HIV-1-infected MDM secretome after exposure to JWH-133. Colors in the tube of isolation of monocytes indicates the separation of blood components (yellow = plasma; blue = lymphocytes, monocytes, and platelets, white = granulocytes, and red = red blood cells). In the tubes of 13 dpi MDM supernatants, green color represents uninfected samples, whereas red color represents HIV-positive samples. Colors in the TMT labeling indicate the 11 different molecular weight tags added to peptide samples prior to mass spectrometry analysis.

Peripheral blood mononuclear cells (PBMCs) were isolated from healthy female donors over 21 years of age in a previous study [15]. The study was managed under the approval of the University of Puerto Rico Medical Sciences Campus Institutional Review Board (IRB) (Protocol #0720116). Informed consent was obtained from each donor according to the Code of Ethics of the World Medical Association and institutional guidelines and regulations. Blood mononuclear cells were cultured in T25 flasks at a concentration of 10 × 10^6^ cells/flask for the isolation of monocyte-derived macrophages (MDMs) after seven days in culture with fetal bovine serum 10% (FBS), human serum 1%, RPMI, and 100 U/mL pen/strep (Sigma; St. Louis, MO, USA). After media were exchanged every three days, on day 7, HIV-1ADA was added to the T25 flasks at an MOI of 0.1, and MDMs were incubated overnight at 37 °C in 5% CO_2_. After cells were washed with media and treated with CB2R agonist JWH-133 at 0.5 µM, half of the medium was exchanged with fresh medium containing JWH-133 every three days. This concentration of JWH-133 was selected based on our previous study, which demonstrated that this was the minimum concentration that induced a significant effect in reducing HIV-1 replication and CATB secretion from HIV-infected MDMs [16]. Supernatants were removed on day 12 post-infection and replaced with serum-free media. After overnight culture, MDM serum-free supernatants from 13 dpi were collected, aliquoted, and frozen at −80 °C (Figure 4).

### 4.2. Preparation of MDM Serum-Free Supernatants for Protein ID and TMT Labeling

The preparation of samples was performed as previously described, with some modifications [15,76,77]. In a sample tube with approximately 30 µg of cell supernatant, 50 µL of 10% sodium dodecyl sulfate (SDS) was added to initiate acetone precipitation. Thereafter, it was heated for 15 min at 70 °C, and cold acetone (~1 mL) was added to the sample up to a final dilution of 15%. Samples were incubated overnight at −20 °C. The next day, samples were centrifuged at 10,000× *g* for 10 min, and, following the removal of supernatants, a sample buffer containing 2X Laemmli buffer + β-mercaptoethanol (Bio-Rad, Hercules, CA, USA) was added. Proteins were rehydrated with sample buffer, heated for 10 min at 70 °C, and run at 150 volts for 20 min in a pre-made gel (Mini-PROTEAN TGX 12%). Gels were stained using Biosafe Coomassie G-250 staining and documented using Chemi-Doc XRS + (Bio-Rad, La Jolla, CA, USA). Coomassie-stained proteome bands were cut manually in 1 mm^3^ cubes. For destaining, a mixture of 50% acetonitrile and 50 mM ammonium bicarbonate was added; thereafter, proteins were reduced using 25 mM dithiothreitol (DTT) in 50 mM ammonium bicarbonate for 30 min at 55 °C and alkylated with 10 mM iodoacetamide (IAA) in 50 mM ammonium bicarbonate for 30 min in the dark. Samples were digested with sequencing-grade modified trypsin (Promega) in 50 mM ammonium bicarbonate at a ratio of 1:50 (trypsin–protein) overnight at a temperature of 37 °C for a maximum of 16 h. The peptides were extracted from the gel pieces using 150 µL of a mixture of 50% acetonitrile and 2.5% formic acid in water and 150 µL of 100% acetonitrile. Digests were dried for subsequent TMT labeling.

### 4.3. Protein ID and TMT Labeling

Protein ID and TMT labeling were performed as previously described [15,76,77]. The TMT labeling of supernatants was performed following the manufacturer’s instructions (Thermo Fisher Scientific, Waltham, MA, USA). A total of 9 samples (30 μg each) were delivered into one TMT10-plex platform, accommodating three donors with their conditions in each platform. TMT reagents were reconstituted in acetonitrile on the same day of use. As specified by the manufacturer’s protocol (Thermo Fisher Scientific), dried extracted samples were reconstituted in 100 mM triethyl ammonium bicarbonate (TEAB), which was the dissolution buffer, and labeled with the TMT10-plex labeling reagents (41 μL, 0.8 mg). The TMT labels were added, followed by one-hour incubation with occasional vortexing and a quenching step of 15 min. Finally, all labeled samples were mixed to generate a final pool that was later subjected to fractionation. The specific TMT tags used for each sample are described in Supplementary Table S8. Fractionation was performed using the Pierce High-pH Reversed-Phase Peptide Fractionation Kit (REF 89875) and following the manufacturer’s instructions (Thermo Fisher Scientific). Briefly, the column was conditioned twice using 300 μL of acetonitrile and centrifuged at 5000× *g* for 2 min, and the steps were repeated using 0.1% trifluoroacetic acid (TFA). The TMT-labeled pool was reconstituted in 300 μL of 0.1% TFA, loaded onto the column, and washed with water and 5% acetonitrile/0.1% triethylamine (TEA). Thereafter, the sample was eluted 8 times into 8 different vials using a series of elution solutions with different acetonitrile/0.1% TEA percentages, as suggested by the manufacturer. Fractions were dried and stored for mass-spectrometric analysis. All 8 fractions generated were run in the mass spectrometer instrument.

### 4.4. Mass Spectrometry

Mass spectrometry analyses were performed as previously described [15,76,77]. A HPLC system (Easy nLC 1200) (Thermo Fisher Scientific) was used for peptide separation. First, the peptides were loaded onto a Pico Chip H354 REPROSIL-Pur C18-AQ 3 µM 120 A (75 µm × 105 mm) chromatographic column (New Objective, Littleton, MA, USA) with a gradient time of 128 min. Separation was obtained at a rate of 300 nL/min as follows: 7–25% 0.1% formic acid in 80% acetonitrile (Buffer B) for 102 min, 25–60% Buffer B for 20 min and 60–95% for 6 min. After separation, the peptides were sprayed and analyzed using a mass spectrometer operated in positive polarity mode and data-dependent mode using a Q-Exactive Plus Orbitrap Mass Spectrometer (Thermo Fisher Scientific). The MS1 (full scan) was measured in the range of 375 to 1400 *m*/*z*, with a resolution of 70,000. To select the ten most intense ions for HCD fragmentation and MS2 (MS/MS) analysis with a resolution of 35,000, a dynamic exclusion parameter was created in 30.0 s with a repeat count of three.

### 4.5. Protein Identification and Quantitative Analyses

MS/MS raw data files were searched using Proteome Discoverer version 2.5 (Thermo Fisher Scientific) with the SEQUEST HT algorithm, and proteins were identified using the human protein database from the same software’s protein knowledge base. Trypsin was included in the search as an enzyme for proteolysis, with a range of 6–144 in peptide length. The precursor mass tolerance was set at 20 ppm, with 0.02 Da as the fragment mass tolerance. Oxidation +15.995 Da (M) was included as a dynamic modification. The static modifications included were carbamidomethyl +57.021 Da (C) and tandem mass tag (TMT) reagents +229.163 Da (any N-terminal end, K). The false discovery rate was set at 0.01 (strict) and 0.05 (relaxed). The raw protein files obtained were exported from the Proteome Discoverer software to .xls format using Microsoft Excel 2016 (CA, USA) for further bioinformatic analysis. Quantitative proteomics analysis was performed using TMT-based LC-MS/MS to identify and quantify proteins secreted by HIV-infected macrophages exposed to the CB2R agonist JWH-133.

### 4.6. Bioinformatics and Statistical Analyses

Following an already developed protocol at the Translational Proteomics Center [15,76,77], we used MetaboAnalyst 5.0 for protein statistical analysis. MetaboAnalyst is a user-friendly, streamlined metabolomics data analysis platform (https://www.metaboanalyst.ca/home.xhtml, accessed on 3 September 2025) [78,79,80] that is also used for statistical analyses of proteomics data [25,78,79,80,81,82]. We determined the statistically differential protein abundance using a single-channel analysis of HIV+MDM-JWH-133 vs. HIV+MDM-vehicle and HIV + vehicle vs. uninfected vehicle. Differentially expressed proteins were considered significant with a fold change cutoff of 1.5 (|Log2 FC| ≥ 0.58) and *p*-value < 0.05. (i.e., *p*-value ≤ 0.05, 95% confidence). We used normalized abundances [25] provided by Yadira Cantres from the Translational Proteomics Center (File name: TMT_Analy-sis_Alana_Mera_26Nov2024_Exp26Nov2024.xlsx). Then, 2D principal component analyses (PCAs) were performed for both comparisons, using the protein concentration as the evaluating variable (PERMANOVA *p*-value (based on 999 permutations) = 0.2, 95% confidence). For the volcano plot, the threshold was set as −log10 *p*-value ≥ 1.30 (or *p*-value ≤ 0.05) and a fold change cutoff of 1.5 (|Log2 FC| ≥ 0.58).

### 4.7. Ingenuity Pathway Analysis

Differentially expressed proteins identified by tandem mass tag (TMT)-based quantitative proteomics were analyzed using Ingenuity Pathway Analysis (IPA; QIAGEN Inc., Redwood City, CA, USA) to investigate canonical pathways, molecular functions, and networks modulated by HIV infection and CB2R activation. A total of seven dysregulated proteins (fold change ≥ |1.5|, *p* ≤ 0.05) identified from the comparisons of HIV + vehicle vs. control and HIV + JWH-133 vs. HIV + vehicle were included. Each protein was annotated for subcellular localization (nucleus, cytoplasm, plasma membrane, or other) and the molecular type (enzyme, transporter, peptidase, or transcription regulator). Analyses were performed using the Ingenuity Knowledge Base (Genes Only) restricted to *Homo sapiens* and immune/macrophage tissues. Canonical pathway overlays were generated through comparison analysis to identify shared and unique pathways between conditions. The statistical significance of pathway enrichment was determined by a right-tailed Fisher’s exact test, and pathways were ranked by −log(*p*) values.

## Figures and Tables

**Table 1:** Unique proteins identified in HIV-infected MDM supernatants.

Protein	Symbol	# Unique Peptides	Molecular Function
Cathepsin B	*CTSB*	12	Lysosomal enzyme that causes proteolysis. Extracellular CTSB secreted by macrophages plays an important role in neuronal death [11,12,13,14,15,16,17,18,19].

# = Number.

**Table 2:** Unique proteins identified in HIV-infected MDM supernatants exposed to JWH-133 agonist.

Protein	Symbol	# Unique Peptides	Molecular Function
Phosphoglycerate kinase 1	*PGK1*	7	Enzyme that catalyzes the reversible transfer of phosphate groups. This is part of glycolysis, a critical energy-producing process.
Alpha-1-antitrypsin	*SERPINA1*	5	Enzyme with many with anti-inflammatory and immunomodulatory properties.
Glyceraldehyde-3-phosphate dehydrogenase	*GAPDH*	7	Enzyme that participates in glycolysis, an energy-producing process.
Heat shock cognate 71 kDa protein	*HSPA8*	5	Molecular chaperone implicated in several cell processes, including the protection of the proteome from stress.
Alpha-actinin-1	*ACTN1*	6	Cytoskeletal activity that helps to maintain the cell shape.
Galectin-3	*IGALS3*	3	Contributes to the regulation of innate and adaptive immunity.
Cathepsin S	*CTSS*	5	Lysosomal enzyme that causes proteolysis in the extracellular space at a neutral pH and plays a key role in neuron regeneration.
Moesin	*MSN*	8	Signal transduction activity or receptor binding; cytoskeletal activity; nucleic acid binding activity.
6-Phosphogluconate dehydrogenase, decarboxylating	*PGD*	7	Produces NADPH, a major reducing protein required for lipid production and protecting the cell against oxidative stress.
Actin-related protein 3	*ACTR3*	5	Cytoskeletal protein, chromatin remodeling.

Immunoglobulin was excluded from this table as it was considered as traces left by Fc receptor binding to MDMs from previous exposure to human serum in the culture media. Identified proteins are represented by their gene symbol. # = Number.

**Table 3:** Differentially abundant proteins identified per group comparison with a fold change ≥ |1.5| and *p*-value ≤ 0.05.

Comparison	Differentially More Abundant	Differentially Less Abundant	Total Differentially Abundant
HIV + Vehicle vs. Control	↑ 5	↓ 2	7
HIV + JWH-133 vs. HIV + Vehicle	0	↓ 2	2

**Table 4:** Differentially expressed proteins secreted by HIV-infected macrophages compared to uninfected controls.

Symbol	Entrez Gene Name	Function in HIV Infection of Macrophages	HIV vs. Uninfected	
			Fold Change	*p*-Value
Q96S97	Myeloid-associated differentiation marker (*MYADM*)	Transmembrane protein that increases with myeloid differentiation and is associated with inflammatory processes. Changes in myeloid differentiation marker levels were reported during inflammation in Rhinovirus asthma [26] and during HIV activation from latency, particularly in macrophages [27,28]. No direct evidence found linking MYADM to HIV infection of macrophages.	1.54	1.46 × 10^−2^
Q96QD8	Sodium-coupled neutral amino acid symporter 2 (*SNAT2* or *SLC38A2*)	Transports small neutral amino acids glutamine and glycine along with sodium ions into cells. Plays vital roles in glutamine–glutamate circulation, synthesis of proteins, and cell growth. Functions as a symporter, co-transports two substances in the same direction across a membrane. No direct evidence found linking SNAT2 to HIV infection of macrophages.	1.68	2.07 × 10^−2^
Q6P179	Endoplasmic reticulum aminopeptidase 2 (*ERAP2*)	Located in the ER lumen and crucial for peptide trimming during antigen processing for major histocompatibility complex (MHC) class I presentation, which is essential in initiating an immune response to infected cells. ERAP2 is correlated with resistance to HIV-1 infection, possibly secondary to its effect on antigen processing and presentation [29]. Secreted ERAP2 from MDMs has been shown to reduce HIV-1 replication in cell cultures by activating monocytes and T cells [30].	1.64	2.56 × 10^−2^
Q96IJ6	Mannose-1-phosphate guanyl transferase alpha (*GMPPA*)	Enzyme that belongs to the family of transferases. They transfer phosphorus-containing nucleotide groups, participating in fructose and mannose metabolism. This process is relevant to HIV because mannose-containing glycans on the HIV envelope (gp120) are essential for binding to host cells, and the HIV mannose receptor 1 (hMRC1) interacts with these glycans, influencing both viral entry and release. While hMRC1 can act as an antiviral factor that restricts HIV particle release, the virus can counteract this by downregulating hMRC1 expression, and the interaction between hMRC1 and Env is often virus isolate-specific [31].	1.66	4.20 × 10^−2^
O75923	Dysferlin (*DYSF*)	Dysferlin is essential for muscle membrane repair and, when mutated, causes a group of genetic muscle diseases. It functions by mediating calcium-dependent vesicle fusion and recruitment to damaged muscle cell membranes. Studies have shown that dysferlin is present in monocytes and macrophages and that dysferlin-deficient macrophages exhibit altered behavior, such as increased phagocytosis [32]. No specific role in HIV-1 infection was found.	1.70	2.59 × 10^−2^
O75563	Src kinase-associated phosphoprotein 2 (*SKAP2*)	Human adaptor protein involved in the Src signaling pathway, B cell and macrophage adhesion, and cytoskeletal organization. Acts as a substrate for Src family kinases (SFKs) and couples the B cell receptor to integrin activation, playing a role in immune cell function. Regulates actin polymerization, which is important for the movement and function of immune cells like T cells and macrophages. HIV-1 protein Nef recruits SFKs to promote viral transcription, and SFKs like Lck facilitate HIV-1 assembly [33]. Src kinase, in its phosphorylated form, is present in and secreted via extracellular vesicles (EVs) in HIV-infected cells. EV-associated c-Src plays a role in reactivating latent HIV-1 by initiating signaling cascades that promote viral gene expression and the remodeling of chromatin [34].	−1.59	3.59 × 10^−2^
Q5VUJ9	Dynein regulatory complex protein 8 (*DRC8* or *EFCAB2*)	Dynein regulatory complex protein 8 (DRC8) is a protein found in various organisms, including humans. A component of the nexin–dynein regulatory complex (N-DRC); a key regulator of ciliary/flagellar motility, which maintains the alignment and integrity of the distal axoneme and regulates microtubule sliding. HIV “hijacks” the dynein transport machinery for microtubule motility, establishing a new model of viral trafficking by directly co-opting host dynein motors [35].	−3.56	4.50 × 10^−2^

**Table 5:** Differentially expressed proteins secreted by HIV-infected macrophages exposed to JHW-133 compared to unexposed controls.

Symbol	Entrez Gene Name	Function in HIV Infection of Macrophages	HIV + Agonist vs. HIV	
			Fold Change	*p*-Value
Q9Y265	RuvB-like 1 (*RVBL1*)	RVBL1 is a component of several important host protein complexes involved in DNA repair and gene regulation. RVBL1/2 is indispensable for pro-inflammatory responses and the antimicrobial activity of macrophages. RUVBL1/2 inhibitors may have therapeutic potential in treating diseases caused by the aberrant activation of pro-inflammatory pathways [36]. RuvB-like 1 (RVBL1) and its homolog, RuvB-like 2 (RVBL2), interact with HIV-1 to regulate viral protein expression and replication. The viral envelope protein (Env) antagonizes RVBL2, which normally inhibits HIV-1 Gag expression. This interaction is crucial for HIV-1 to balance viral protein production, control viral RNA stability, and produce infectious virion particles. In HIV-1-positive patients, RVBL2 levels positively correlate with viral loads and disease progression status. These findings reveal a mechanism by which HIV-1 regulates its protein expression [37]. While HIV-1 can hijack exosomal machinery for the trafficking of viral RNA and proteins, infected cells can secrete exosomes containing viral components like the Env protein. The specific role of RVBL1 in this context has not been reported [38].	−1.91	3.52 × 10^−2^
Q86YZ3	Hornerin (*HRNR*)	Hornerin is an S100 protein family member that plays a key role in calcium binding. Hornerin is involved in normal and pathological cell functions including gene transcription, inflammatory and immune responses, the regulation of protein phosphorylation, transcription factors, antimicrobial responses, Ca^2+^ homeostasis, and the dynamics of cytoskeleton constituents, as well as cell proliferation, differentiation, and death. Inhibitors of specific S100 proteins are currently being developed as therapeutics for diseases including diabetes mellitus, heart failure, neurological diseases, and several types of cancer [39]. It has been reported that biotinylated HRNR can be incorporated into HIV-Gag virus-like particles [39].	−2.15	3.88 × 10^−2^

**Table 6:** Functional annotation of differentially expressed proteins using Ingenuity Pathway Analysis (IPA).

Comparison	ID	Symbol	Entrez Gene Name	Location	Type(s)
HIV+ Vehicle vs. Control	O75923	*DYSF*	Dysferlin	Plasma membrane	Other
	Q5VUJ9	*EFCAB2*	EF-hand calcium binding domain 2	Other	Other
	Q6P179	*ERAP2*	Endoplasmic reticulum aminopeptidase 2	Cytoplasm	Peptidase
	Q96IJ6	*GMPPA*	GDP-mannose pyrophosphorylase A	Cytoplasm	Enzyme
	Q96S97	*MYADM*	Myeloid-associated differentiation marker	Nucleus	Other
	O75563	*SKAP2*	Src kinase-associated phosphoprotein 2	Cytoplasm	Other
	Q96QD8	*SLC38A2*	Solute carrier family 38 member 2	Plasma membrane	Transporter
HIV+ JWH-133 vs. HIV + Vehicle	Q86YZ3	*HRNR*	Hornerin	Cytoplasm	Other
	Q9Y265	*RUVBL1*	RuvB-like AAA ATPase 1	Nucleus	Transcription regulator

## Data Availability

Most of the data generated or analyzed during this study are included in this published article. All proteomics raw datasets generated in the current study have been deposited in the ProteomeXchange [83] Consortium via PRIDE [84], a partner repository, with the following dataset identifier: Project Name: Cannabinoid Receptor Type 2 Agonist JWH-133 Stimulates Antiviral Factors and Decreases Proviral, Inflammatory, and Neurotoxic Proteins in HIV-Infected Macrophage Secretome; Project accession: PXD069890; Project DOI: 10.6019/PXD069890 [83,84].

## Supplementary Materials

The following supporting information can be downloaded at: https://www.mdpi.com/article/10.3390/ijms262110596/s1.
