# Supplementary material for: Cannabinoid Receptor Type 2 Agonist JWH-133 Stimulates Antiviral Factors and Decreases Proviral, Inflammatory, and Neurotoxic Proteins in HIV-Infected Macrophage Secretome
Source: Int J Mol Sci. 2025 Oct 30;26(21):10596. doi: 10.3390/ijms262110596 (PMC12608856; doi:10.3390/ijms262110596)
Supplement: Supplementary file 1 [file ijms-26-10596-s001.zip › Supplementary Table S7. Common proteins identified that were also found in TMT analyses but did not meet the significance criteria for differentiall.pdf]

Supplementary Table S7. Common proteins identified that were also found in TMT analyses but did not meet the significance criteria for differentially abundant proteins between control and experimental groups.

P02647	Apolipoprotein A-I
P02649	Apolipoprotein E
P02786	Transferrin receptor protein 1
P02787	Serotransferrin
P07339	Cathepsin D
P07355	Annexin A2
P08670	Vimentin
P09467	Fructose-1,6-bisphosphatase 1
P13796	Plastin-2
P14618	Pyruvate kinase PKM
P14780	Matrix metalloproteinase-9
P36222	Chitinase-3-like protein 1
P60709	Actin, cytoplasmic 1

*Green color depicted common proteins found in ID and TMT experiments. These proteins did not meet the quantitative proteomics significance criteria of Fold Change ≥ |1.5| and *p*-value ≤ 0.05.
