# Supplementary material for: Cannabinoid Receptor Type 2 Agonist JWH-133 Stimulates Antiviral Factors and Decreases Proviral, Inflammatory, and Neurotoxic Proteins in HIV-Infected Macrophage Secretome
Source: Int J Mol Sci. 2025 Oct 30;26(21):10596. doi: 10.3390/ijms262110596 (PMC12608856; doi:10.3390/ijms262110596)
Supplement: Supplementary file 1 [file ijms-26-10596-s001.zip › Supplementary Table S8. TMT Experimental Design.pdf]

Supplementary Table S8. TMT Experimental Design

TMT Label	Sample ID	Treatment
126	D1 Uninfected (-)	Vehicle (Veh)
127N	D1 HIV+	Vehicle (Veh)
127C	D1 HIV+	JWH-133 0.5uM (J 0.5 μM)
128N	D8 Uninfected (-)	Vehicle (Veh)
128C	D8 HIV+	Vehicle (Veh)
129N	D8 HIV+	JWH-133 0.5uM (J 0.5 μM)
129C	D10 Uninfected (-)	Vehicle (Veh)
130N	D10 HIV+	Vehicle (Veh)
130C	D10 HIV+	JWH-133 0.5uM (J 0.5 μM)
