# Supplementary figures and images for: Cannabinoid Receptor Type 2 Agonist JWH-133 Stimulates Antiviral Factors and Decreases Proviral, Inflammatory, and Neurotoxic Proteins in HIV-Infected Macrophage Secretome
Source: Int J Mol Sci. 2025 Oct 30;26(21):10596. doi: 10.3390/ijms262110596 (PMC12608856; doi:10.3390/ijms262110596)

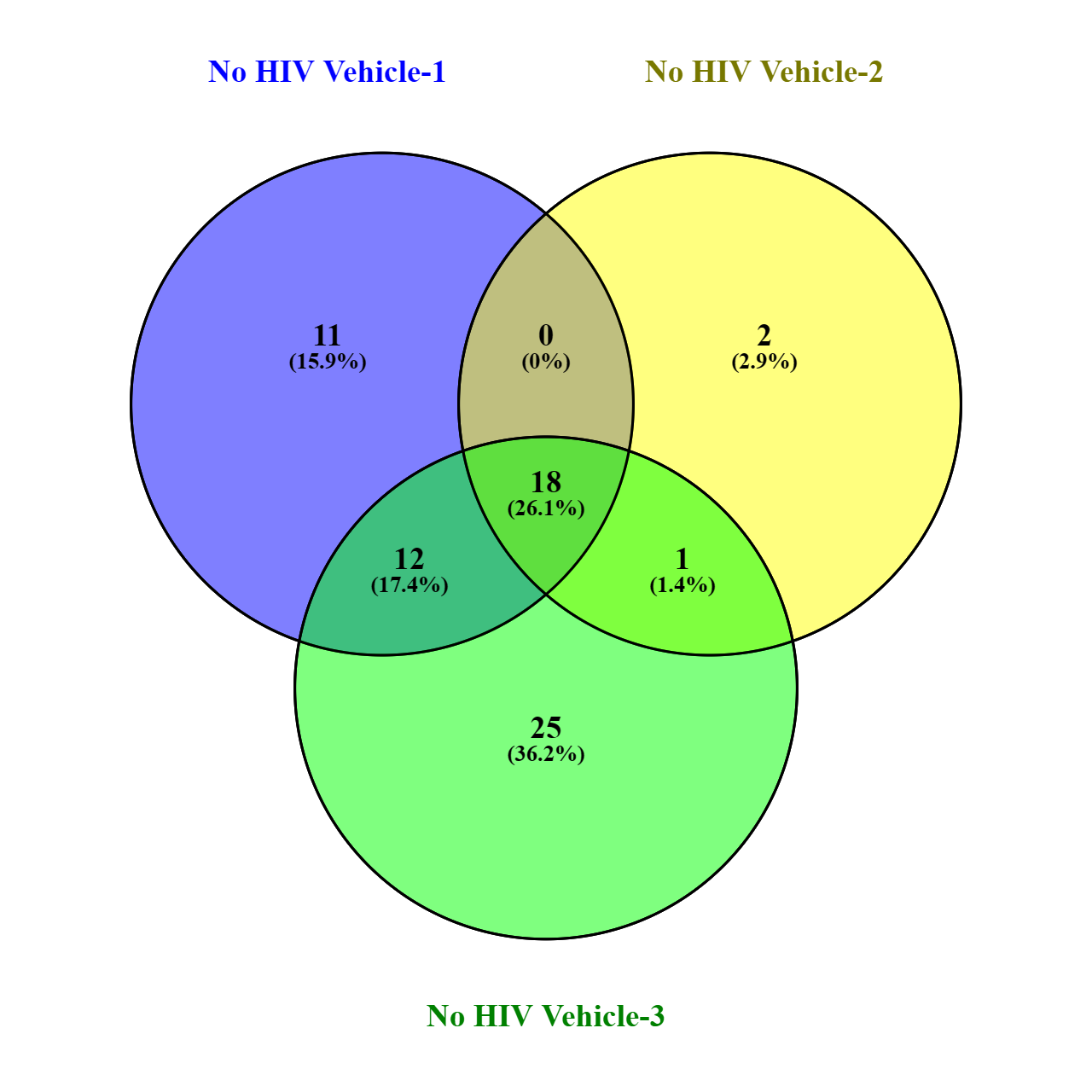

Supplement: Supplementary file 1 [file ijms-26-10596-s001.zip › Supplementary Figure S1. Venn Diagram_Uninfected Vehicle - Replica.png]

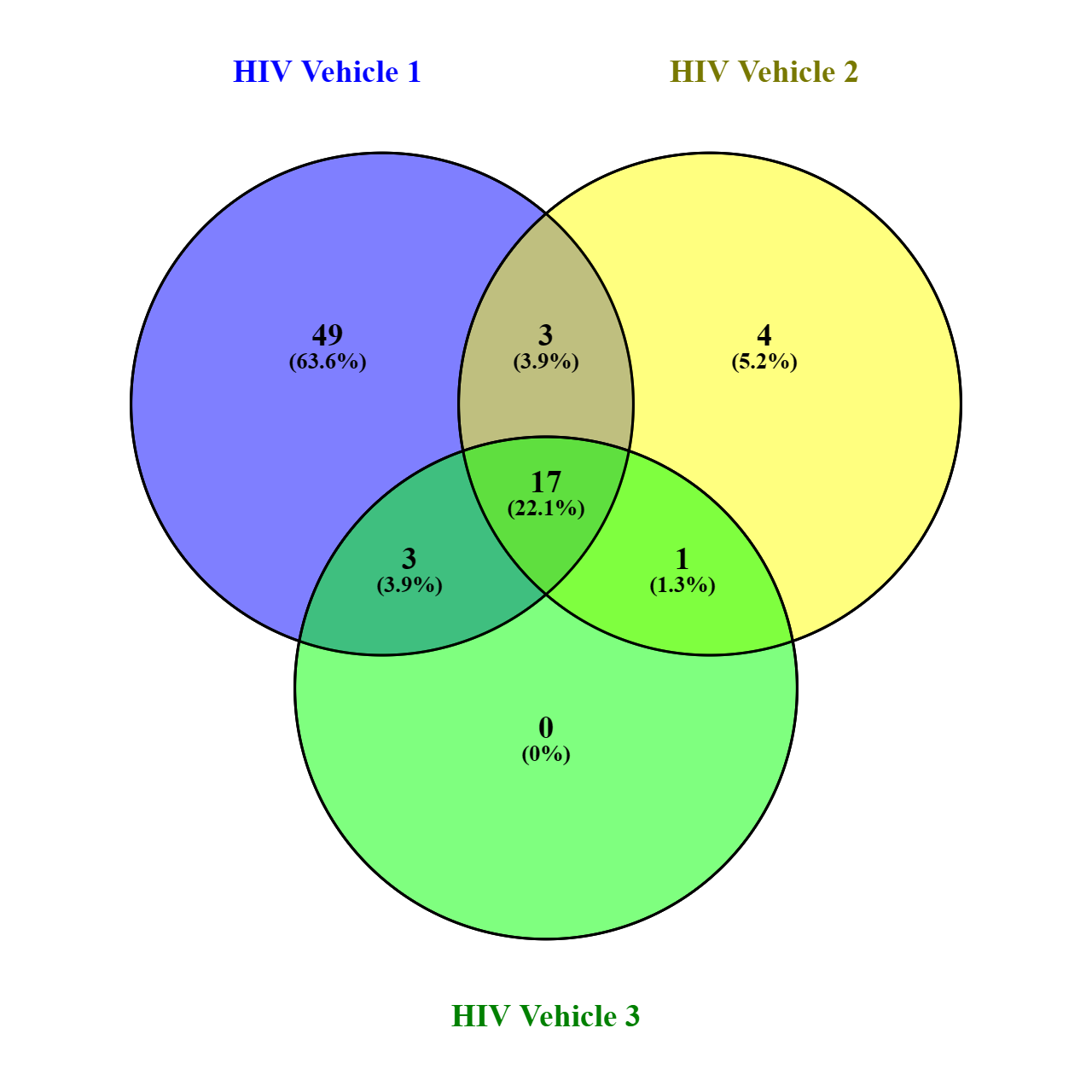

Supplement: Supplementary file 1 [file ijms-26-10596-s001.zip › Supplementary Figure S2. Venn Diagram_HIV Vehicle - Replica.png]

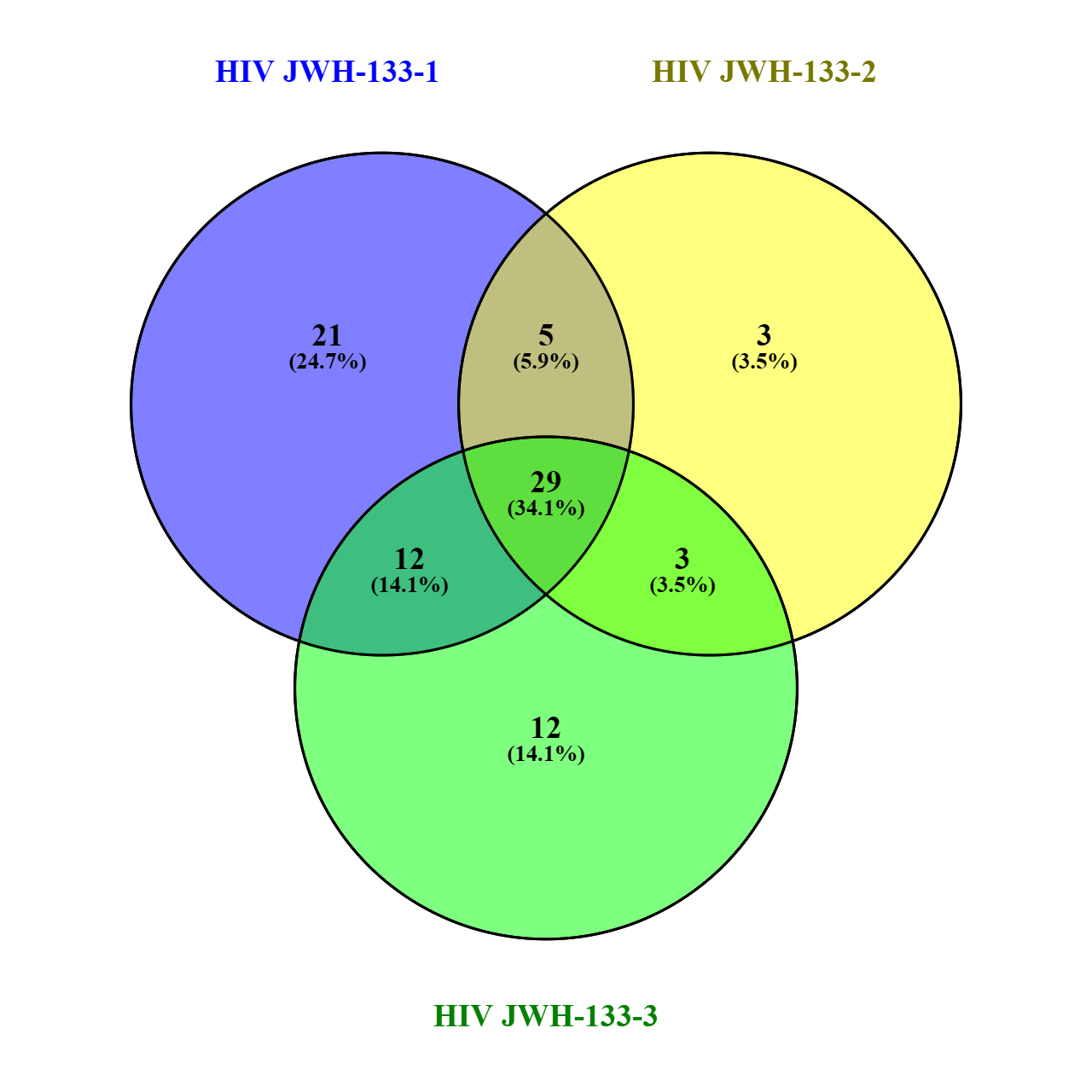

Supplement: Supplementary file 1 [file ijms-26-10596-s001.zip › Supplementary Figure S3. Venn Diagram_HIV JWH-133 - Replica.png]
